# Peptide Mix from *Olivancillaria hiatula* Interferes with Cell-to-Cell Communication in *Pseudomonas aeruginosa*

**DOI:** 10.1155/2019/5313918

**Published:** 2019-09-24

**Authors:** Edward Ntim Gasu, Hubert Senanu Ahor, Lawrence Sheringham Borquaye

**Affiliations:** ^1^Central Laboratory, Kwame Nkrumah University of Science and Technology, Kumasi, Ghana; ^2^Department of Chemistry, Kwame Nkrumah University of Science and Technology, Kumasi, Ghana

## Abstract

Bacteria in biofilms are encased in an extracellular polymeric matrix that limits exposure of microbial cells to lethal doses of antimicrobial agents, leading to resistance. In *Pseudomonas aeruginosa*, biofilm formation is regulated by cell-to-cell communication, called quorum sensing. Quorum sensing facilitates a variety of bacterial physiological functions such as swarming motility and protease, pyoverdine, and pyocyanin productions. Peptide mix from the marine mollusc, *Olivancillaria hiatula*, has been studied for its antibiofilm activity against *Pseudomonas aeruginosa.* Microscopy and microtiter plate-based assays were used to evaluate biofilm inhibitory activities. Effect of the peptide mix on quorum sensing-mediated processes was also evaluated. Peptide mix proved to be a good antibiofilm agent, requiring less than 39 *μ*g/mL to inhibit 50% biofilm formation. Micrographs obtained confirmed biofilm inhibition at 1/2 MIC whereas 2.5 mg/mL was required to degrade preformed biofilm. There was a marked attenuation in quorum sensing-mediated phenotypes as well. At 1/2 MIC of peptide, the expression of pyocyanin, pyoverdine, and protease was inhibited by 60%, 72%, and 54%, respectively. Additionally, swarming motility was repressed by peptide in a dose-dependent manner. These results suggest that the peptide mix from *Olivancillaria hiatula* probably inhibits biofilm formation by interfering with cell-to-cell communication in *Pseudomonas aeruginosa.*

## 1. Introduction

Drug resistance in human health and agriculture is a major hurdle in efforts towards the achievement of Goals 2 and 3 of the Sustainable Development Goals—achieving zero hunger and good health and well-being for all [1–3]. Due to indiscriminate use of antibiotics in aquaculture, poultry, and livestock farming, the incidence of antibiotic resistance is on the rise. The incidence of antibiotic resistance could increase the cost of meat and fish production as new drugs will be required to manage hitherto uncomplicated infections. Additionally, the yield of these food produces could potentially plummet and thus threaten global efforts in achieving food sufficiency [4]. Meanwhile, many antibiotics used to treat various life-threatening and debilitating human infections have lost their efficacy, and a return to the pre-antibiotic era is on the horizon. Antibiotics are rapidly losing their efficacy primarily due to extensive, unrestricted, and often inappropriate use of most antibiotics [5]. The presence of sub-standard drugs on the markets of many developing countries also contributes to the loss of efficacy of antibiotics and hence antibiotic resistance [6]. Healthcare costs are on the rise due to antibiotic resistance, and the economic impact of morbidity and mortality due to failed treatments as a result of antibiotic resistance is enormous [7–10]. Pathogenic microbes utilize a variety of strategies to overcome the action of antimicrobial agents. These include the development or acquisition of resistance genes, formation of specialized persister cells, alteration of antimicrobial agent and/or antimicrobial target sites, the use of efflux strategies, and biofilm formation, amongst others [11–13]. Unfortunately, these pathogens seem to have outpaced our capacities to manage them.

Biofilm formation, where microorganisms secrete a covering of extracellular polymeric substance (EPS) around them, is one of many strategies utilized by bacteria to diminish the effects of antibiotics and thus establish resistance. Bacteria within biofilms benefit from protection against host immune systems and are shielded from lethal antibiotic doses [14–16]. *Pseudomonas aeruginosa* (*P. aeruginosa*) is a model biofilm-forming bacteria and an opportunistic pathogen implicated in cystic fibrosis-related respiratory infections and chronic infections in immunocompromised patients [17]. Due to its ability to produce proteases, *P. aeruginosa* contributes largely to host tissue degradation as well as meat and protein-rich food spoilage [17–19]. The expression of genes associated with biofilm formation in *P. aeruginosa* is controlled by cell-to-cell communication, referred to as quorum sensing (QS), and multiple studies have shown that a strain of *P. aeruginosa* defective in QS is unable to form biofilm [20–23]. Additionally, QS has been shown to interfere with the expression of various *P. aeruginosa* virulence factors such as pyoverdine, pyocyanin, and proteases. QS also mediates surface motility, growth inhibition, and antibiotic production [24]. As such, the QS machinery in *P. aeruginosa* has been suggested as a plausible target for the development of therapeutic agents [25].

Strategies for biofilm treatment include prevention of microbial surface attachment, inhibition of biofilm development by killing early surface-colonizing bacteria with biofilm-forming potential, interference with quorum sensing, and eradication of mature biofilms [26]. Various natural products capable of attenuating biofilm formation and inhibiting QS have been reported in the literature [23, 27]. A class of compounds that have shown promise in interfering with QS-mediated processes such as biofilm formation are peptides [10, 28, 29]. Antimicrobial peptides (AMPs) function by targeting microbial cell membrane, binding with DNA to inhibit protein synthesis and detoxifying lipopolysaccharides. AMPs have been shown to be active even against multidrug-resistant microorganisms [30]. The exploration of AMPs could potentially expand the available options for eradicating bacterial biofilms.

Many marine invertebrates survive ocean surface biofouling and microbial attack by relying solely on their innate immune system which is principally composed of peptides. These peptides are secreted at very high concentrations in order to escape dilution by surrounding seawater and enhance efficacy. AMPs from these sources can be target specific, charged, and amphipathic [31] and could therefore be utilized as potential agents against biofilm-forming bacteria. This work sought to explore the biofilm inhibitory capabilities of the peptide mix extracted from the marine mollusc, *Olivancillaria hiatula*, and evaluate the potential of the peptide mix to interfere with some QS-controlled processes in *P. aeruginosa*.

We herein report that the peptide mix from *Olivancillaria hiatula* inhibits biofilm formation in *P. aeruginosa* and eradicates pre-formed biofilm as well. Additionally, the peptide mix inhibits the expression of some virulence factors such as pyoverdine, pyocyanin, and proteases at sub-lethal doses and has a significant impact on the swarming motility of *P. aeruginosa.* Together, these results suggest that the peptide mix attenuates biofilm formation by interfering with cell-to-cell communication in *P. aeruginosa.*

## 2. Materials and Methods

### 2.1. Chemicals

All chemicals were of analytical grade and were purchased from Sigma-Aldrich, St. Louis, MO, USA, unless stated otherwise. Gentamicin was prepared in sterile MilliQ water.

### 2.2. Sample Collection and Identification

Molluscs were collected from the shores of a beach at Eikwe (4°58′00″ N 2°28′47″ W), in the Western Region of Ghana, and kept on ice. Samples were then transported to the Department of Chemistry, Kwame Nkrumah University of Science and Technology (KNUST), Kumasi, and stored in a refrigerator at 4°C. Organism was identified at the Department of Fisheries and Marine Sciences, University of Ghana, Legon, as *Olivancillaria hiatula*.

### 2.3. Peptide Extraction

The peptide mix was obtained by the method described by Sathyan with some modification [32]. In iced state, shells were removed and the whole body tissue was pulverized. Tissue slurry was added to 10% (v/v) acetic acid and kept for 12 hours at 4°C. The mixture was centrifuged to remove debris, retaining supernatant (acetic acid digest). Ice-cold acetone (25 mL) was then added to the supernatant while shaking, and this was kept at 4°C for 24 hours to facilitate peptide precipitation. The precipitates were collected by centrifuging at 5000 rpm for 15 minutes. Precipitates were then frozen at −80°C. Nitrogen gas was used to blow out traces of solvents after freezing at −80°C. The peptides were reconstituted in 25% acetonitrile (ACN) prepared in 0.1% trifluoroacetic acid (TFA) to give 5 mg/mL stock solution and stored at 4°C prior to use [32, 33].

### 2.4. Characterization of Peptide Mix by Infrared Spectroscopy

The infrared spectrum of the peptide mix was acquired using a Fourier transform infrared equipment (UATR Two, PerkinElmer, Waltham, MA, USA) by scanning the regions between 4000 cm^−1^ and 400 cm^−1^ followed by baseline correction.

### 2.5. Bacterial Strain and Growth Conditions

*Pseudomonas aeruginosa* ATCC 4853 was obtained from the Department of Pharmaceutics, Faculty of Pharmacy and Pharmaceutical Sciences of the College of Health Sciences, KNUST. Unless indicated otherwise, the bacteria were grown at 37°C on nutrient agar or in nutrient broth (Oxoid, United Kingdom). Bacteria colonies on nutrient agar were used to prepare colony suspensions in sterile saline, adjusted to 0.5 McFarland standard, and further diluted in sterile double-strength nutrient broth to give ∼2 × 10^5^ CFU/mL.

### 2.6. Minimum Inhibitory Concentration

The minimum inhibitory concentration (MIC) of the peptide extract and gentamicin was determined by the broth dilution method described by Wiegand and coworkers with some modification [34]. Twofold serial dilutions of the peptide mix or standard gentamicin were prepared to obtain a concentration range of 2.5 to 4.88 × 10^−3^ mg/mL in a 96-well polypropylene microtiter plate (Thermo Scientific, UK). Overnight culture of *P. aeruginosa* was adjusted to 0.5 McFarland standard in sterile saline and subsequently inoculated in double-strength nutrient broth to an inoculum size of ∼2.0 × 10^5^ CFU/mL. Fifty microliters of inoculum was added to each well to a total volume of 100 *μ*L and incubated at 37°C for 24 hours. MTT (3-(4,5-dimethylthiazol-2-yl)-2,5-diphenyltetrazolium bromide) was added to each well and incubated for 30 minutes. The MIC was determined as the lowest concentration of peptide extract that inhibited growth of test organism which was indicated by the absence of purple coloration after incubation. Sub-MICs were concentrations at which the growth of bacteria was not affected after 24 hours. All tests were performed in triplicate.

### 2.7. Bacterial Growth Curve

The growth of *P. aeruginosa* (∼2 × 10^5^ CFU/mL) in the presence of MIC and sub-MICs of peptides was evaluated by optical density (OD) measurements. Briefly, microplates were prepared with serial dilutions of the peptides and bacteria (as for the MIC assay) and incubated at 37°C with 3 seconds of shaking, followed by OD (600 nm) measurements every hour for 24 hours (BioTeK® Synergy H1 Multimode Microplate Reader, Germany). Growth curves of OD_600_ measurements against time were plotted.

### 2.8. Evaluation of Biofilm-Forming Ability of *P. aeruginosa*

Petri plates containing glass slides were sterilized and inoculated with 8 mL of double-strength nutrient broth containing ∼2 × 10^5^ CFU/mL *P. aeruginosa* overnight culture and incubated at 37°C for 24 hours. Glass slides were washed with sterile water, dried, and stained with 1% crystal violet. Excess crystal violet was washed with deionized water, glass slides were dried, and micrographs were obtained with a light microscope (Leica Model CME Microscope, Buffalo, NY, USA) using immersion oil. Images were captured from different fields [35].

### 2.9. FT-IR Characterization of Extracellular Polymeric Substance (EPS)

Test tubes containing 8 mL (∼2 × 10^5^ CFU/mL) bacteria were incubated at 37°C without agitation, to allow biofilm formation. After 48 hours of incubation, the dense slimy matrix formed at the media-air interface was decanted and washed with 30% acetic acid and ice-cold acetone [36]. The infrared spectrum of the EPS was acquired as described previously.

### 2.10. Inhibition of Biofilm Formation

In order to estimate the minimum biofilm inhibition concentration, biofilm formation assay was performed [27, 37]. Microtiter plates containing bacteria with or without peptides at various concentrations were incubated for 24 hours at 37°C without agitation. Plates were then washed with deionized water to remove unattached bacteria and stained with 0.1% crystal violet followed by absorbance measurements at 595 nm (BioTeK® Synergy H1 Multimode Microplate Reader, Germany). Percentage biofilm inhibition was estimated from the normalized OD values using the following equation:

(1) $$\begin{array}{c} \%\text{ inhibition}=\frac{\text{control}-\text{treated}}{\text{control}}\times 100. \end{array}$$

### 2.11. Inhibition of Biofilm Formation on Glass Slides

Petri plates containing glass slides were sterilized and inoculated with 8 mL of double-strength nutrient broth containing ∼2 × 10^5^ CFU/mL *P. aeruginosa* overnight culture with or without peptide treatment and incubated at 37°C for 24 hours. Glass slides were washed with sterile water, dried, and stained with 1% crystal violet. Excess crystal violet was washed with deionized water, glass slides were dried, and micrographs were obtained with a light microscope (Leica Model CME Microscope, Buffalo, NY, USA) using immersion oil. Images were captured from different fields.

### 2.12. Eradication of Preformed Biofilm on Glass Slides

Petri plates containing glass slides were sterilized and inoculated with 8 mL of double-strength nutrient broth containing ∼2 × 10^5^ CFU/mL *P. aeruginosa* overnight culture and incubated at 37°C for 24 hours. The plates, which had glass slides with preformed biofilms, were then treated with 2.5 mg/mL of peptide mix prepared in sterile double-strength nutrient broth and incubated for a further 24 hours. Glass slides were then washed with sterile water, dried, and stained with 1% crystal violet. Excess crystal violet was washed with deionized water, glass slides were dried, and micrographs were obtained with a light microscope (Leica Model CME Microscope, Buffalo, NY, USA) using immersion oil. Images were captured from different fields.

### 2.13. Pyoverdine Quantification

*P. aeruginosa* inoculum was incubated in the absence (growth control) and presence of sub-MIC (1/2 MIC, 1/4 MIC, 1/8 MIC, 1/16 MIC, and 1/32 MIC) doses of gentamicin and peptide extract at 37°C for 48 hours. Culture media were then centrifuged at 4,000 rpm for 45 minutes. One hundred microliters of cell-free supernatant was dispensed into a 96-well microtiter plate for pyoverdine measurement. The relative concentration of pyoverdine in all treated supernatants with respect to control (no drug) was measured by fluorescence (BioTeK® Synergy H1 Multimode Microplate Reader, Germany) at an excitation wavelength of 405 nm and an emission wavelength of 465 nm [27, 38]. Percentage inhibition was determined relative to untreated culture (control) by the expression in (1).

### 2.14. Pyocyanin Quantification

*P. aeruginosa* was incubated as described in the pyoverdine inhibition assay. Cell-free supernatants were collected after centrifugation at 4000 rpm for 45 minutes. Four milliliters of chloroform was then added to 8 mL of the supernatant and vortexed 10x for 2 seconds (green-blue chloroform sinks to the bottom of the tube). Samples were then centrifuged for 2 minutes at 4000 rpm, and the supernatant on top of the green-blue chloroform was decanted. Three milliliters of 0.2 M HCl was then added to each tube and vortexed 10x for 2 s and then centrifuged for 2 minutes at 4000 rpm. Supernatant (pink layer) was transferred into a cuvette and absorbance measured at 520 nm [39]. Pyocyanin concentration (*μ*g/mL) was calculated by multiplying the absorbance value at 520 nm with 17.072 (molar extinction coefficient of pyocyanin at 520 nm). Percentage inhibition was determined relative to untreated culture (control) by the expression in (1).

### 2.15. Protease Expression Assay

*P. aeruginosa* inoculum was incubated in the absence (growth control) and presence of sub-MIC (1/2 MIC, 1/4 MIC, 1/8 MIC, 1/16 MIC, and 1/32 MIC) doses of standard antibiotic gentamicin and peptide mix at 37°C for 48 hours. Culture media were transferred into centrifuge tubes and then centrifuged at 4000 rpm for 45 minutes. The amount of L-tyrosine released from the degradation of casein by proteases in the cell-free supernatant was evaluated using standard protocols as described elsewhere [40]. The amount of L-tyrosine released by proteases expressed in the untreated cultures was used to estimate the percentage inhibition of protease expression by (1).

### 2.16. Swarming Motility Assay

Swarming motility was investigated in treated and untreated cultures of *P. aeruginosa* using a swarming motility media composed of 0.8% nutrient broth, 0.5% nutrient agar, and 0.5% glucose. The media surface was briefly dried, and bacterial cells from overnight cultures treated with or without peptide mix were gently inoculated using a sterile toothpick at the center of the agar surface and incubated at 37°C for 24 hours and 48 hours. The diameter of the circular pattern was measured [41].

### 2.17. Data Analyses

All data analyses and graphs were done using GraphPad Prism version 6.0 for Windows (GraphPad Software, San Diego, CA, USA) and Microsoft Excel 2013. Data values of experimental results were recorded as the mean ± standard deviation. Where necessary, significance was determined by one-way ANOVA.

## 3. Results

### 3.1. Infrared Characterization

**Figure 1:**
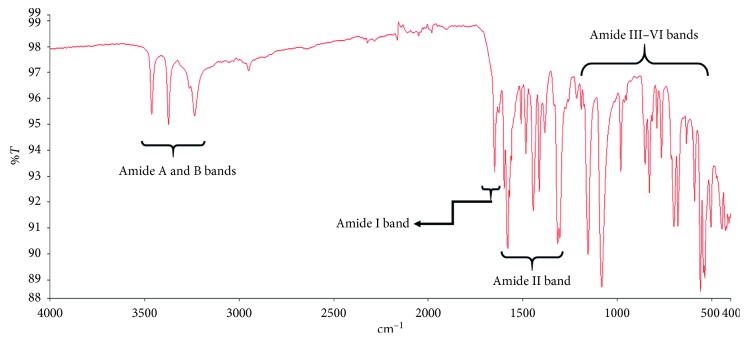
Fourier transform infrared (FTIR) spectrum of the peptide mix isolated from *Olivancillaria hiatula*. Peak characteristics of the amide A and B bands (3100–3500 cm^−1^), amide I band (1600–1700 cm^−1^), amide II band (1480–1600 cm^−1^), and amide III–VI bands (500–1300 cm^−1^) have been shown, as described elsewhere [42, 43].

The infrared spectrum of the peptide mix isolated from *Olivancillaria hiatula* is shown in Figure 1. The spectrum was consistent with that of an archetypal peptide. Peaks representative of –N–H, C=O, and –C–H stretching and bending vibrations were visible in the spectrum.

### 3.2. Minimum Inhibitory Concentration

In order to assess the effect of peptide mix on biofilm formation, peptide concentrations that were to be evaluated need to have little or no effect on the viability of the microbes. Thus, sub-MIC doses were to be used in the assays. The minimum inhibitory concentration (MIC) of the peptide mix on *P. aeruginosa* was evaluated and found to be 39.06 *μ*g/mL. The MIC of gentamicin was also determined and was found to be 1.95 *μ*g/mL.

### 3.3. Bacterial Growth Rate and Growth Curves

**Figure 2:**
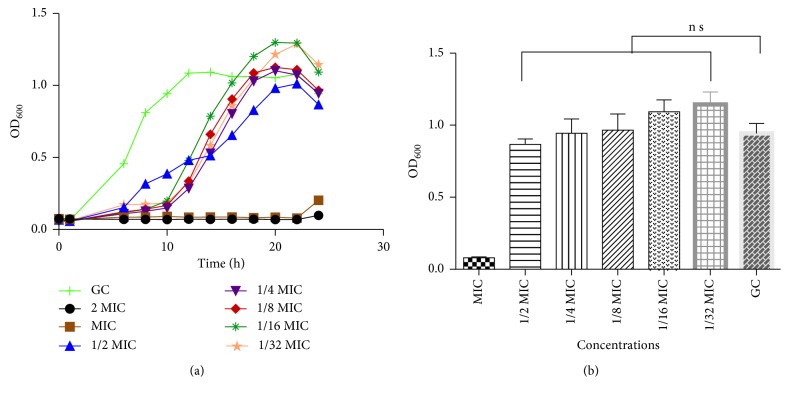
(a) Growth curve of *Pseudomonas aeruginosa* ATCC 4853 in the absence (GC) and presence of varying concentrations of peptide mix (MIC, 1/32 MIC). After 24 hours of growth, *Pseudomonas aeruginosa* absorptions (OD_600_) were similar, with no significant difference between all treated cells relative to the untreated control. (b) Cell density of *Pseudomonas aeruginosa* ATCC 4853 cultivated in the absence (GC) and presence of sub-MICs of peptide mix. Cell density was recorded after 24 hours of growth. Mean values of 3 independent experiments and their standard deviations are shown. When compared with the untreated control (GC), no significant difference (*P* < 0.05) was observed. Cell density for MIC treatment was significantly different (*P* < 0.0001) from that of sub-MIC-treated groups (MIC, minimum inhibitory concentration; GC, growth control).

Quorum sensing is population-dependent; thus, to estimate interference, bacterial growth in the presence or absence of peptide mix at MIC and sub-MICs was examined using 96-well plate based OD_600_ measurements (Figure 2(a)). The log and stationary phases in the growth control (GC) began at 5 and 12 hours, respectively. At the MIC, a lag time of 16 to 18 hours was observed with decreased absorbance at log or stationary phase. At sub-MICs, the lag time was prolonged to ∼10 hours and log phase to ∼23 hours with no observable stationary phase as observed in the growth control. After 24 hours (Figure 2(b)), there was no significant difference in OD_600_ absorptions, indicating that bacteria cells did grow to about the same extent, in spite of the differences in growth pattern between treated and untreated groups.

### 3.4. Biofilm Formation and Characterization

**Figure 3:**
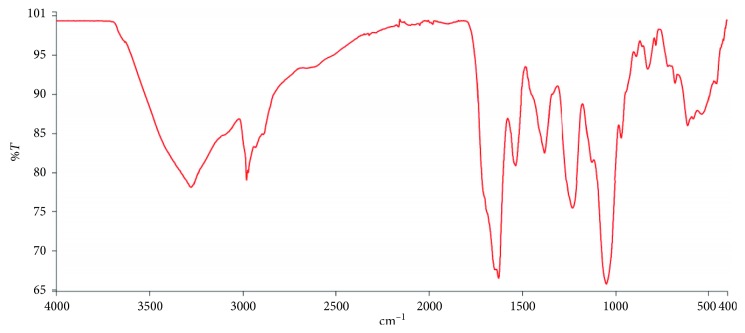
FTIR spectrum of the EPS of *Pseudomonas aeruginosa* ATCC 4853. The EPS matrix was precipitated from air-media interface and contains absorption bands arising from N–H and O–H stretches in proteins and sugars (3280–3450 cm^−1^), –C–H– stretches in lipids and fatty acids (2850–2980 cm^−1^), amide carbonyl (–C=O) stretches in proteins (1540–1630 cm^−1^), as well as O-acetyl (–C–O–C–) stretches in polysaccharides and nucleic acids (900–1380 cm^−1^) [36, 44].

The *Pseudomonas aeruginosa* ATCC 4853 strain used in this study proved to be ideal for this study as biofilm formation was observed on both glass slides and in microtiter plates. The EPS of the biofilm formed was also characterized by infrared spectroscopy and the spectrum obtained (Figure 3) showed peaks largely consistent with that of a biofilm matrix. There were absorption bands arising from –N–H and –O–H stretches in proteins and sugars (3280–3450 cm^−1^), –C–H– stretches in lipids and fatty acids (2850–2980 cm^−1^), amide carbonyl (–C=O) stretches in proteins (1540–1630 cm^−1^), as well as O-acetyl (–C–O–C–) stretches in polysaccharides and nucleic acids (900–1380 cm^−1^). The spectrum was consistent with those published elsewhere [36, 44].

### 3.5. Effect of Peptide Mix on Biofilm Formation

**Figure 4:**
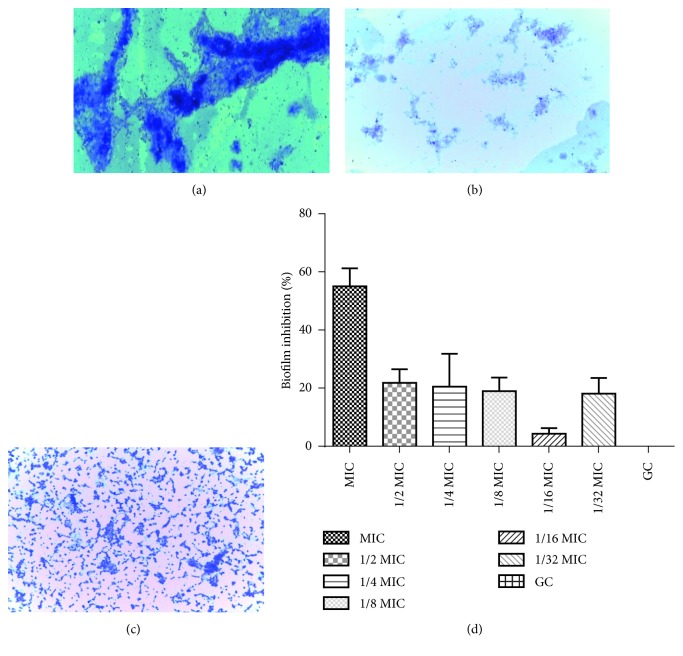
Antibiofilm activity of peptide mix. Micrographs of *P. aeruginosa* biofilm on glass slides: (a) untreated control showing dense biofilm in the absence of peptide, (b) biofilm inhibition in the presence of 1/2 MIC of peptide mix, and (c) disruption of preformed biofilm treated with 2.5 mg/mL of peptide mix. (d) Antibiofilm effect of peptide concentration on *Pseudomonas aeruginosa*. Each bar represents mean ± SD of triplicate experiments in a microtiter plate-based assay.

To evaluate the effects of peptides on biofilm formation, OD measurements and microscopy were used. Light microscopy revealed strong biofilm-forming ability in the control group (Figure 4(a)) owing to high absorption of the crystal violet dye. The treated group (Figure 4(b)) showed markedly reduced crystal violet-stained grooves in the micrograph, with fewer crystal violet stains in the 1/2 MIC-treated setup. The peptide mix also disrupted pre-formed biofilm on the glass slides as observed by the disruption of biofilm in the treated group relative to the control (Figure 4(c)). The microtiter plate-based assay was also used to determine the effect of peptide mix on biofilm formation. At the MIC, biofilm formation was inhibited to about 55%. This reduced to 22% at 1/2 MIC and dropped to a low of 4% at 1/16 MIC. However, inhibition increased to 18% at 1/32 MIC (Figure 4(d)).

### 3.6. Effect of Peptide Mix on Secretion of Virulence Factors

**Figure 5:**
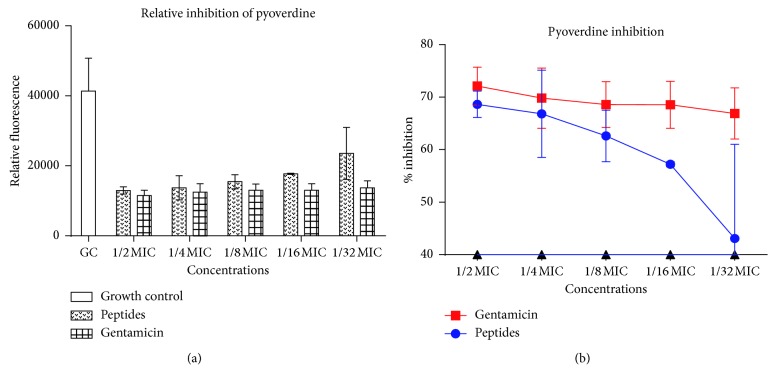
Pyoverdine inhibition. (a) Relative fluorescence of pyoverdine secreted by *Pseudomonas aeruginosa* with and without sub-MIC doses of the peptide mix and standard drug, gentamicin. Each bar represents mean ± SD of fluorescence intensities of 3 independent experiments. (b) Percentage inhibition of pyoverdine secretion in *Pseudomonas aeruginosa* in the presence of sub-MIC doses of the peptide mix and gentamicin. Percentage inhibitions were computed with respect to the fluorescence of the control group. Each bar represents mean ± SD of triplicate experiments.

**Figure 6:**
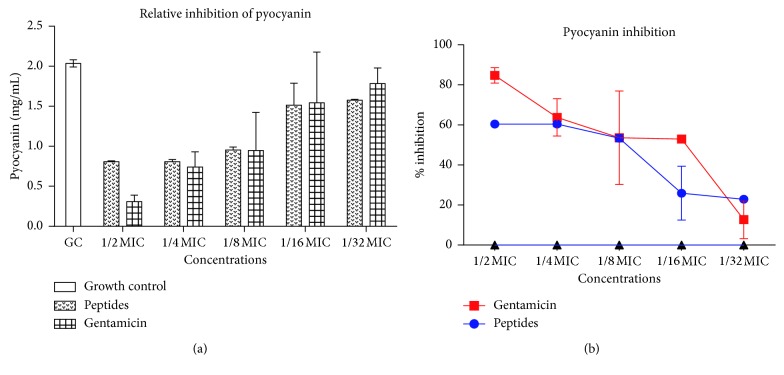
Pyocyanin inhibition. (a) Pyocyanin production by *Pseudomonas aeruginosa* with and without sub-MIC doses of the peptide mix and standard drug, gentamicin. Each bar represents mean ± SD of pyocyanin levels in 3 independent experiments. (b) Percentage inhibition of pyocyanin secretion in *Pseudomonas aeruginosa* in the presence of sub-MIC doses of the peptide mix and gentamicin. Percentage inhibitions were computed with respect to the control group. Each bar represents mean ± SD of triplicate experiments.

**Figure 7:**
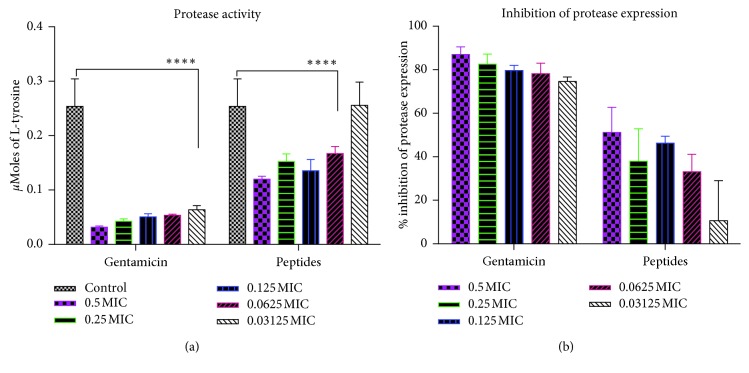
Protease inhibition. (a) Levels of tyrosine produced from casein digestion by proteases in cell-free supernatants of treated and untreated cultures of *Pseudomonas aeruginosa.* Treated cultures were prepared in the presence of sub-MIC doses of the peptide mix and standard drug, gentamicin. Each bar represents mean ± SD of tyrosine concentration in 3 independent experiments. (b) Percentage inhibition of protease expression in *Pseudomonas aeruginosa* in the presence of sub-MIC doses of the peptide mix and gentamicin. Percentage inhibitions were computed with respect to the control group. Each bar represents mean ± SD of triplicate experiments.

Since QS mediates the expression of genes responsible for the production and secretion of virulence factors in *P. aeruginosa*, we sought to evaluate the ability of the peptide mix to interfere with the production of pyoverdine, pyocyanin, and proteases by *P. aeruginosa.* Gentamicin is a known QS inhibitor and so was used as a positive control [27]. Relative fluorescence of pyoverdine in treated cultures showed that pyoverdine production was reduced in a largely dose-dependent manner. At 1/2 MIC and 1/32 MIC, gentamicin reduced pyoverdine production by 72% and 66%, respectively, whereas the peptide mix reduced pyoverdine production by 69% and 43%, respectively (Figure 5). For pyocyanin production, 85% and 13% reductions were observed for gentamicin at 1/2 MIC and 1/32 MIC, respectively, whereas 61% and 23% reductions were observed for peptide mix at similar concentrations. Pyocyanin inhibition in both cases was similar at 1/2 MIC and 1/8 MIC (Figure 6). Untreated cell-free supernatants evaluated for the presence of proteases yielded elevated levels of protease activity owing to their ability to digest casein and release large amounts of amino acids. Levels (*μ*moles) of L-tyrosine estimated from a standard curve were used to assess the extent of protease inhibition in untreated and treated cultures. In gentamicin cultures, there was 87.06% to 74.60% inhibition of protease expression compared to untreated cultures. When treated with peptide mix, cultures gave moderate inhibition of protease expression—51.21% and 10.63%, at 1/2 MIC and 1/32 MIC, respectively (Figure 7).

### 3.7. Effect on Swarming Motility

**Figure 8:**
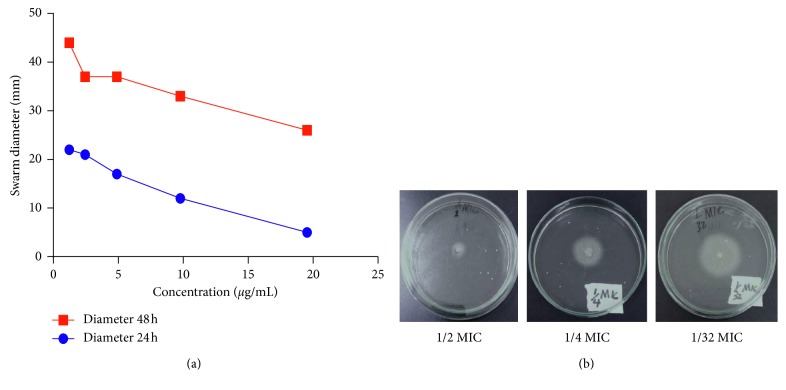
Inhibition of swarming motility. (a) Line plot of *Pseudomonas aeruginosa* swarm diameter (mm) on an agar plate. Each well was seeded with bacteria and peptide mix (sub-MIC doses of the peptide mix) and incubated for 24 hours (blue line) or 48 hours (red line). Each point is the mean of triplicate experiments. (b) Representative pictures of swarming motility of *Pseudomonas aeruginosa* in the presence of 1/2 MIC, 1/4 MIC, and 1/32 MIC doses of the peptide mix.

There was a marked reduction in swarming motility of *P. aeruginosa* treated with peptide mix. Reduction in motility was also dose-dependent. After 24 hours, swarm diameters varied between 5 mm and 22 mm for peptide-treated culture inoculums. This increased after 48 h of incubation to between 26 mm and 44 mm (Figure 8).

## 4. Discussions

Bacteria that exist in their biofilm state have been found to be more virulent than their planktonic counterparts. These bacteria secrete a hydrated matrix that consists of polysaccharides, proteins, nucleic acids, and lipids. Biofilm-associated bacteria often cause infections that are difficult to treat, primarily due to their multidrug-resistant nature. New and effective molecules to treat such infections are therefore urgently needed. There is increasing evidence about the involvement of QS in biofilm formation, maintenance, and dispersal [45]. QS controls the expression of genes that are responsible for biofilm formation, growth control, production of virulence factors, and motility in *P. aeruginosa* [24]. Quorum sensing inhibitors (QSIs) have therefore been proposed as potential antibiofilm agents [22, 27, 45]. Antimicrobial peptides have shown great promise as potential therapeutics for infectious disease control, and a number of them are currently in advanced clinical trials [26, 30, 46]. The peptide mix from *Olivancillaria hiatula* has shown interesting antimicrobial potential [47] and was therefore evaluated for its ability to attenuate biofilm formation and interfere with other QS-mediated processes in the model biofilm-forming pathogenic organism *P. aeruginosa*.

The peptide mix from *Olivancillaria hiatula* was obtained via acetone precipitation of an acetic acid extract. The infrared (IR) spectrum of the extract revealed characteristic amide I absorptions at about 1650 cm^−1^. This is due to carbonyl (–C=O) stretching vibrations of the amide functionality. The amide II absorptions observed from 1480 to 1575 cm^−1^ were prominent and are due to –N–H bending and –C–N stretching vibrations. The absorptions between 3200 and 3500 cm^−1^ represent the amide A and B bands and are due to –N–H stretching vibrations. Finally, there were peaks consistent with amide III–VI regions (500–1300 cm^−1^) in the spectrum. The infrared spectrum therefore showed a sample rich in peptides [42, 43, 48].

The *P. aeruginosa* strain used in this study proved to be a very good biofilm-forming microbe. The micrograph obtained after crystal violet staining showed deep, violet grooves indicative of a biofilm. The infrared spectrum of the EPS matrix precipitated from the biofilm is consistent with that studied by other researchers [36, 44]. The results indicated the presence of a mixture of macromolecules such as proteins, nucleic acids, lipids, and polysaccharides.

Due to the antimicrobial action of peptides isolated from molluscs [26, 32, 33], we evaluated the MIC of the peptide mix against *P. aeruginosa.* An MIC of 39 *μ*g/mL indicates a good antibacterial agent. An MIC dose, however, will completely inhibit the growth of the bacteria and thus prevent biofilm formation, motility, or virulent factors' expression. Additionally, since biofilm formation, virulent factors' production, and swarming motility are all dependent on bacteria quorum size, it was important to show that the sub-MIC of peptides to be used did not inhibit bacterial growth. We thus monitored bacterial growth kinetics at OD_600._ There was no significant difference in the OD_600_ absorption between untreated group (growth control) and the sub-MIC-treated cells (Figure 2(a)). While there were differences in the rate of growth, the OD_600_ after 24 hours clearly showed that bacteria growth was uninhibited (Figure 2(b)) and quorum sizes could be attained. This indicates that any effect on any of the processes investigated does not occur as a result of attenuation of bacteria growth, but rather due to interference of peptide mix in important cellular processes.

Sub-MIC doses of the peptide mix were evaluated for their ability to modulate biofilm formation in *P. aeruginosa.* Biofilm inhibition was in the range of 4–22%. Analysis of our data showed that peptide concentrations between the MIC and 1/2 MIC will be required to inhibit biofilm formation by about 50%. The micrographs obtained from the glass slide-based assay show the reduction in biofilm formed on the glass slide from the treated culture in comparison with that of the control. The microtiter assay is thus complementary to the glass slide-based assay. However, the microtiter plate-based assay provides a means for quantification. The peptide mix was also able to scatter pre-formed biofilm. The eradication of pre-formed biofilm required a much higher concentration of peptide mix (2.5 mg/mL) to observe any effect. This suggests that the peptide mix probably functions by interfering with the biofilm formation process, rather than removing biofilm that has already been formed. It has been suggested that antibiofilm peptides function by preventing microbes from adhering to surfaces, killing early surface colonizers, killing preformed biofilm-associated cells, and inhibiting the quorum sensing machinery of the microbe [49]. We postulated that the peptide mix from *Olivancillaria hiatula* probably interferes with cell-to-cell communication in *P. aeruginosa* and thus inhibits biofilm formation via this route.

Since QS in *P. aeruginosa* also controls motility and the expression of virulence factors such as pyocyanin, pyoverdine, and proteases [24], peptide mix should also interfere with the expression and production of these virulent factors as well as the motility of the microorganism. We therefore evaluated the levels of pyocyanin, pyoverdine, and proteases expressed by *P. aeruginosa* in the presence and absence of sub-MIC doses of the peptide mix. We also examined the effect of these peptide concentrations on *P. aeruginosa* swarming motility. Pyoverdine levels were reduced in the presence of the peptide mix, with inhibitions occurring in an essentially dose-dependent manner. At 1/2 MIC, pyoverdine production was inhibited by over 60%. For pyocyanin, production was inhibited by about 61% at 1/2 MIC, whereas over 50% reduction in protease expression was also observed. These results show that the peptide mix from *Olivancillaria hiatula* does indeed interfere with the production of virulent factors, and the same mechanism is used in biofilm inhibition.

The basic *P. aeruginosa* siderophore, pyoverdine, is able to both sequester iron from host depots and act as a QS signaling molecule. Iron-bound pyoverdine interacts with the *P. aeruginosa* cell receptor FpvA, and this complex in turn interacts with the antisigma factor, FpvR, causing the upregulation of exotoxin A, an endoprotease, and of pyoverdine itself [50]. Pyocyanin induces oxidative stress in host and promotes the secretion of airway mucus. It has also been reported that *P. aeruginosa* secretes proteases as a virulent factor to progress pathogenesis [50]. All these virulent factors are also important for biofilm development and maintenance and contribute significantly to the devastating nature of *P. aeruginosa* infections.

We also investigated the effect of peptides on swarming motility. Cells treated with peptide mix showed less swarming motility when compared to the untreated control. Similar to the effects shown by peptide mix on virulent factors, a dose-dependent relationship was observed. Swarming is an intricate communal behavior developed by microbes due to several environmental signals, and this facilitates microbial motility on a semisolid surface and is used in the colonization of host tissues. Swarming motility in *P. aeruginosa* is flagella-driven, and this phenomenon is controlled by QS. Swarming motility also positively influences surface attachment—the 1^st^ step in microbial biofilm formation [27, 41].

The ability of the peptide mix from *Olivancillaria hiatula* to inhibit biofilm formation in *P. aeruginosa* as well as swarming motility and the expression of virulent factors therefore suggests that the mode of action involves the inhibition of a common factor in all these processes. Since QS controls all these activities in *P. aeruginosa*, the peptide mix probably interferes with cell-to-cell communication. Very few drugs on the market inhibit both quorum sensing and biofilm formation [27]. Identification of compounds and extracts that perform both dual functions limits the likelihood for the formation of antimicrobial resistance and provides a facile strategy for control of pathogenic microbes. The promise shown by the peptide mix from *O. hiatula* in this regard provides an opportunity for developing novel therapeutics to target pathogenic bacteria.

## 5. Conclusion

In summary, the results from the present study shows that the peptide mix from *Olivancillaria hiatula* is a potent antibiofilm agent that functions probably by attenuating QS in *P. aeruginosa.* Identification of the peptide sequence will facilitate unambiguous establishment of the mode of action. Efforts in this regard are currently underway.

## Data Availability

All data generated or analyzed during this study are included in this manuscript.
